# The Optimisation of Bitter Gourd-Grape Beverage Fermentation Using a Consolidated Response Surface Methodology (RSM) and Artificial Neural Network (ANN) Approach

**DOI:** 10.3390/plants12193473

**Published:** 2023-10-04

**Authors:** Tintswalo Lindi Maselesele, Tumisi Beiri Jeremiah Molelekoa, Sefater Gbashi, Oluwafemi Ayodeji Adebo

**Affiliations:** 1 Food Innovation Research Group, Department of Biotechnology and Food Technology, Faculty of Science, University of Johannesburg, P.O. Box 17011, Johannesburg 2028, South Africa; tintswalom38@gmail.com; 2 Department of Biotechnology and Food Technology, Faculty of Science, University of Johannesburg, Doornfontein Campus, P.O. Box 17011, Johannesburg 2028, South Africa; tumisim@uj.ac.za (T.B.J.M.); sefatergbashi@gmail.com (S.G.)

**Keywords:** ANN, bitter gourd beverage, fermentation, optimisation, RSM

## Abstract

The present study adopted a response surface methodology (RSM) approach validated by artificial neural network (ANN) models to optimise the production of a bitter gourd-grape beverage. Aset of statistically pre-designed experiments were conducted, and the RSM optimisation model fitted to the obtained data, yielding adequately fit models for the monitored control variables R^2^ values for alcohol (0.79), pH (0.89), and total soluble solids (TSS) (0.89). Further validation of the RSM model fit using ANN showed relatively high accuracies of 0.98, 0.88, and 0.82 for alcohol, pH, and TSS, respectively, suggesting satisfactory predictability and adequacy of the models. A clear effect of the optimised conditions, namely fermentation time at (72 h), fermentation temperature (32.50 and 45.11 °C), and starter culture concentration (3.00 *v*/*v*) on the total titratable acidity (TTA), was observed with an R^2^ value of (0.40) and RSM model fit using ANN overall accuracy of (0.56). However, higher TTA values were observed for samples fermented for 72 h at starter culture concentrations above 3 mL. The level of 35% bitter gourd juice was optimised in this study and was considered desirable because the goal was to make a low-alcohol beverage.

## 1. Introduction

The twenty-first century has brought with it a new set of challenges in the alcohol industry, as the industry is now expected to adapt to new consumer expectations as well as changes in grape composition and its qualities because of climate change. New frontiers in beverage formulations, as well as consumer demand for lower alcohol content and fruitier aromas as well as new raw materials are some of the current challenges facing the industry [1].

Bitter gourd (*Momordica charantia*), also known as balsam pear, karela, and bitter melon, is an important functional vegetable used to produce low-alcohol, ‘healthy’ beverages [2,3]. This is due to its array of beneficial bioactive compounds such as antioxidants, vitamins, dietary fibre, and minerals [4]. As a result of its antitumour, anti-inflammatory, and antimicrobial properties, bitter gourd is used to control type II diabetes, ‘purify the blood’, induce a bowel movement, and cure the disease of the spleen and liver, gout, and rheumatism [5].

The documentation of low alcoholic beverages from bitter gourd is not widely available despite its growing popularity and increased demand by consumers, a fact that is attributed to changes in consumers’ lifestyles. In addition, there is limited research showing the combination of suitable fruits with bitter gourd to make a palatable beverage with medicinal properties [5]. A few attempts to optimise the fermentation process using response surface methodology (RSM) in order to improve the nutritional and functional properties of bitter gourd beverage have been reported [2,6,7]. Deshaware et al. [6] adopted response surface methodology (RSM) for the optimisation of temperature (31–55 °C), pectinase concentration (4.04–15.92 mL kg^−1^), and incubation time (48–191 min), while Naveen and Joshi [7] used the same technique to produce a low-alcohol beverage from bitter gourd and apple by standardising the concentration of apple juice (20–40%), diammonium hydrogen phosphate (DAHP) (0.050–0.15%), and inoculum size (2.5–7.5%). Likewise, Devaki and Premavalli [2] optimised the development of bitter gourd-fermented beverages using RSM. RSM was used to try to optimise the fermentation process with regard to curd concentration and duration, with a focus on nutritional and functional factors.

Recently, the use of RSM in conjunction with nonlinear multivariate techniques has become standard practice in the beverage industry [8,9]. Specifically, artificial neural networks (ANN) can be used to approximate nonlinear interactions of factors in fermentation processes [10,11]. Since ANNs mimic the brain’s function, they can learn, adapt, solve new problems from ‘experience’, and make relatively accurate predictions depending on the adequacy of the training process [12,13]. Thus, the current study aimed to use an RSM approach validated by ANNs in experimental design to produce a high-quality bitter gourd beverage [14,15].

## 2. Materials and Methods

**Bitter gourd raw material processing**. Fresh bitter gourd (*Momordica charantia*) procured locally in (Mpumalanga Province, South Africa) was sorted, washed thoroughly in running water to remove adhering foreign materials, cut on both ends, and thereafter blended (Milex, Sandton, South Africa). Grapes procured locally at Food Lovers Market were washed and blended using a juicer (Milex, Sandton, South Africa).

**Response surface methodology design of experiments (DoE)**. The suitable ranges for inputs (Table 1 used in the DoE were obtained from the literature and subsequent preliminary experiments. Design-Expert software version 11.0.0 (Stat-Ease Inc., Minneapolis, MN, USA) was used to generate 20 experimental runs (Table 1). 

Blended bitter gourd and grapes were mixed at different concentrations and monitored for pH, alcohol, total titratable acidity (TTA), and total soluble solids (TSS), and we concluded that, at a concentration of 35% bitter gourd and 65% grapes, the ratio would be suitable for enough sugar content for fermentation to take place as well as enough bitter gourd juice for its physiochemical properties to be investigated. The mixture was subsequently inoculated with *Saccharomyces cerevisiae* and *Metschnikowia pulcherimma* (Anchor Yeast, Lallemand, South Africa). Experiments were conducted in triplicates, and samples were withdrawn after each experimental run to test for alcohol (°P), pH, total titratable acidity (TTA) (% lactic acid), and total soluble solids (TSS) (g/100 g).

**Determination of alcohol**. A semi-quantification of alcohol content in the beverage was determined using a digital refractometer for brewing (Hanna Instruments (Pty) Ltd., Johannesburg, South Africa) by placing 1 mL of the beverage on the sample well and observing the reading. The Plato (°P) readings were recorded afterwards.

**Determination of pH**. A pH meter (Hanna Instruments (Pty) Ltd., Johannesburg, South Africa) was first calibrated with standard buffers of pH 4 and 7 and used to measure the pH of respective samples.

**Determination of total titratable acidity (TTA)**. TTA was determined using the AACC Method 02-31.011. This entailed dissolving 10 g of the sample in 100 mL of distilled water. The solution was mixed, and a drop of 1% phenolphthalein was added. The prepared solution was titrated with 0.1 N sodium hydroxide until a faint pink colour was observed.

**Determination of total soluble solids (TSS)**. The TSS value was determined using a digital refractometer (Hanna Instruments (Pty) Ltd., Johannesburg, South Africa) by placing the beverage on the sample well and observing the reading.

**Function fitting and neural network construction**. The input and output data from the RSM experiments were exported to MATLAB R2020a (MathWorks, Natick, MA, USA) software, and a basic code was used to design and run the neural network. A feed-forward neural network with an input layer and an output layer was used. Fermentation time—X_1_ (h), fermentation temperature—X_2_ (°C), and starter culture concentration—X_3_ (%) were used as the neural network inputs, while alcohol content—Y_1_, pH—Y_2_, TTA—Y_3_, and TSS—Y_4_ were used as the neural network outputs. Input and output data from RSM (Table 2) was randomly divided for training (70%), validation (15%), and testing (15%). The Levenberg–Marquardt (LM) training algorithm was used to train, validate, and test the neural network until the desired coefficient of correlation (R2) was obtained.

**Statistical analysis**. All experiments were conducted in triplicates and expressed as mean ± standard deviation. Analysis of variance (ANOVA) was employed to determine the significance of the data using Design-Expert^®^ software version 11.0.0 (Stat-Ease Inc., Minneapolis, MN, USA). Significant F tests at (*p* < 0.05) levels of probability are reported.

## 3. Results

Subsequent to determining optimum conditions, the relationship between inputs and responses was assessed using a second-order optimisation model based on the central composite design (CCD). The effects of inputs on responses are shown in Table 2.

**The effect of input factors on alcohol**. Nonfermented beverage had an alcohol content of 1.10 °P (Table 2). The lowest alcohol content was observed in the uninoculated beverage, fermented for 72 h at 32.50 °C. For the same fermentation period, an alcohol content of 11.70 °P was observed when the fermentation temperature and the starter culture concentration were increased to 45.11 °C and 3.00 *v*/*v*, respectively. Temperature had a significant linear and quadratic effect on alcohol content, as expressed in Equation (1):

(1) $$Y_{1}=3.84154+0.0139719X_{1}+1.91644X_{2}+0.235307X_{3}-0.675X_{1}X_{2}-\;0.125X_{1}X_{3}-0.15X_{2}X_{3}+0.063876{X_{1}}^{2}+1.90235{X_{2}}^{2}-0.130578{X_{3}}^{2},$$

**The effect of input factors on pH**. A low pH value was observed for beverage samples fermented for 72 h, at 32.50 °C. For shorter fermentation times and lower fermentation temperatures, lower pH values were observed when the starter culture concentration was considerably increased (Table 2). Mazlan et al. [16] observed a rapid decrease in pH during the first 16 h of fermentation. The fermentation time, temperature, and starter culture concentration had significant linear effects, while only the fermentation time and temperature had a significant quadratic effect on the pH. An increase in inoculum concentration may lead to an increase in pH values [17].

**The effect of input factors on TTA**. TTA is an important measurement to monitor the progress of an acid-producing fermentation [18]. Anaerobic respiration of lactic acid-producing bacteria may lead to a pH reduction and an increase in TTA during the fermentation period [4]. A clear effect of fermentation time, temperature, and starter culture concentration on the TTA could not be established (Table 2). However, higher TTA values were observed for samples fermented for 72 h at starter culture concentrations above 3.00 *v*/*v*.

**The effect of input factors on TSS**. The highest TSS value (11.30 g/100 g) was observed at a fermentation time of 72 h, a fermentation temperature of 45.11 °C, and a starter culture concentration of 3.00 *v/v* (Table 2). Samples fermented at 40 °C had significantly higher TSS values, while those fermented at lower temperatures had lower TSS values (Table 2). A high TSS value is attributed to solutes leaching into the fermentation media [4]. In addition, the bitter gourd sap and soluble solutes from the shreds may be responsible for the high TSS [19]. The fermentation temperature had a significant quadratic effect on TSS (Table 2).

Multi-response optimisation

Analysis of variance (ANOVA) statistically validated generated solutions, while the best fit of the generated models was examined using the F-value (Table 3). The probability of significance was expressed by *p*-values, with low *p*-values indicating an adequate model in combination with a nonsignificant lack of fit [20]. The lack of fit and the coefficient of determination values (R^2^) define the model’s adequacy [21]. Furthermore, R^2^ reflects the extent of variation in the mean obtained by each model. R^2^ > 0.88 (88%) showed high confidence in predicting the usability of the generated model [22]. The model *p*-values for alcohol, pH, and TSS were 0.02, 0.00, and 0.00, respectively, with F-values greater than 4 (Table 3). The lack of fit was thus insignificant at values over 97% confidence level. The model for TTA was insignificant with a *p*-value of 0.67.

The models for alcohol, pH, and TSS had R^2^ values of 0.79, 0.89, and 0.89, respectively, thus showing their adequacy, accuracy, and predictability. Models with R^2^ values above 70% are favourable since they have a high capability in predicting responses to the evaluated process. The prediction accuracy of these models may be significantly improved by transforming the responses, reducing the complexity of the mode, and/or considering outliers [23]. The mathematical solutions of the generated models are described using polynomial equations (Equations (2)–(4)).

(2) $$Y_{2}=3.71652+0.0319516X_{1}+0.0342816X_{2}+0.0319516X_{3}-0.0125X_{1}X_{2}+\;0.0125X_{1}X_{3}-0.0125X_{2}X_{3}+0.0657492{X_{1}}^{2}+0.0834268{X_{2}}^{2}-\;0.00496152{X_{3}}^{2},$$

(3) $$Y_{3}=6.86128-0.0622378X_{1}-0.0908625X_{2}-0.00998462X_{3}+0.15X_{1}X_{2}+\;0.225X_{1}X_{3}+0.1X_{2}X_{3}+0.100041{X_{1}}^{2}+0.206107{X_{2}}^{2}-0.0590585{X_{3}}^{2},$$

(4) $$Y_{4}=3.82875-0.197707X_{1}+2.04891X_{2}-0.0795488X_{3}-0.425X_{1}X_{2}+\;(1.65029^{-16})X_{1}X_{3}-0.05X_{2}X_{3}+0.2273{X_{1}}^{2}+1.42938{X_{2}}^{2}-0.0732204{X_{3}}^{2}$$

where X1—fermentation time; X2—fermentation temperature; X3—starter culture concentration; Y1—alcohol; Y2—pH; Y3—TTA; Y4—TSS.

**Generated validation using artificial neural network (ANN)**. The Levenberg–Marquardt (LM) training algorithm used in this study is a fast-training algorithm, especially for loss functions [24]. As shown in Table 3, the algorithm revealed high accuracy in training, validating, and testing the ANN inputs and responses. The use of the LM training algorithm resulted in obtaining lower mean squared error (MSE) values. An increase in MSE values causes the LM algorithm to automatically stop generalisation. This is also consistent with a study by Jang et al. [25], which demonstrated that the MSE and MBR, or dataset obtained for the optimisation of fungi co-fermentation for improving anthraquinone content and antioxidant activity using artificial neural networks, were lower than those from the ANN model. In addition, since few parameters and instances were investigated, the algorithm showed better performance and overcame the problem of overfitting [17]. This is also exemplified in a study by Zheng et al. [26], which looked at the production of two antitumour benzoquinones in optimised wheat germ fermentation conditions where an ANN with 11 neurons in the hidden layer yielded the lowest mean square error (MSE) for the validation of dataset and test dataset. The correlation between inputs and responses was determined by monitoring the coefficient of correlation (R^2^). R^2^ = 0 describes a random relationship, and R^2^ = 1 describes a precise relationship.

The mean squared error (MSE) measures the average squared difference between inputs and responses, with lower values indicating a better fit. MSE values are the simplest error functions in ANNs since they highly predict model responses accurately [27]. The overall accuracy of the models was evaluated using both R^2^ and MSE values [28]. The overall R^2^ values for alcohol, pH, TTA, and TSS were 0.98, 0.88, 0.56, and 0.82, respectively (Table 4). Since these values are closer to 1, the models have high reliability in prediction and validation accuracy. In contrast, the TTA model had a 56% reliability in prediction and validation accuracy, as shown by an overall R^2^ value of 0.56. Interestingly, the alcohol model had R^2^ values of 0.99 for training, validation, and testing, indicating a 99% efficiency in estimating outputs (Table 4) [29].

**Figure 1:**
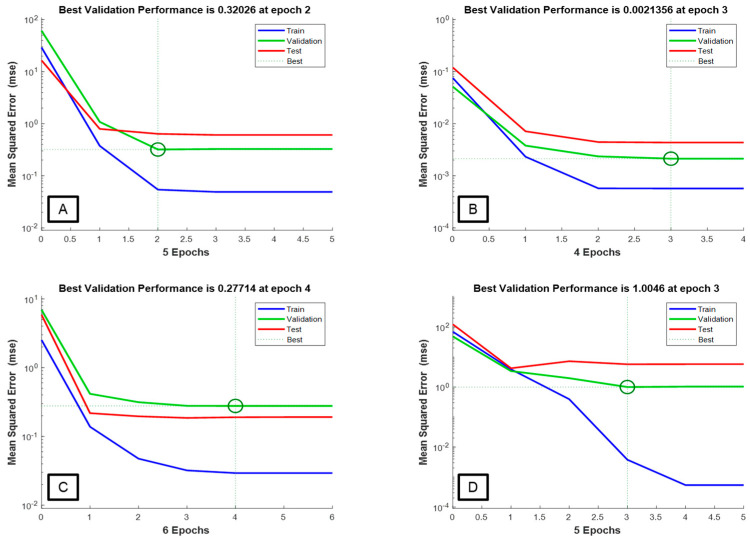
Performance curves for neural network models: alcohol—(**A**); pH—(**B**); TTA—(**C**); TSS—(**D**).

Performance curves that plot MSE values against the number of training of one cycle through the full training dataset are used to evaluate the network’s learning capability by showing the learning direction and incremental training process. Performance curves also unveil unrepresentative validation datasets underfitting and overfitting models, and problems with unrepresentative training datasets [30]. The training performance curves for alcohol, pH, TTA, and TSS are shown in Figure 1. A low error accompanied by a few epochs (iterations) indicates excellent learning capabilities. The ANN models for TTA, TSS, alcohol, and pH had repetitions of 6, 5, 5, and 4, respectively. These are desired since overfitting the training data may occur with an increase in the number of training iterations [31]. A stable decrease in the training and validation MSE values, as well as a significant difference between these values, show a good fit for all the models [32].

## 4. Discussion

In food analysis, the relationship between titratable acidity and pH is reciprocal and deals with acidity. Both parameters are calculated analytically and provide insight into food quality in their unique way. While pH is important for evaluating a microorganism’s ability to flourish in a particular food, titratable acidity is a better predictor of how organic acids in food affect flavour [33].

Due to a loss of acidity, the increase in pH is paralleled by a decrease in titratable acidity [34]. The relationship between the two was observed in the study whereby pH was of low values and TTA remained relatively higher throughout the experiment (Table 1).

Temperature plays a significant role in pH measurements. As the temperature rises, molecular vibrations increase, and as a result, water can ionise and form more hydrogen ions. The presence of more hydronium ions will decrease the pH, due to the fact that there are more hydrogen cations, which make the solution more acidic [35].

High temperature resulted in low pH, thus creating favourable conditions during the fermentation processes for microorganisms to thrive; these conditions have a direct correlation with TSS catalysing the sugars, thus resulting in high TSS values. In accordance with Table 2, the higher the TSS, the higher the alcohol produced; thus, the decrease in TSS is directly proportional to the alcohol content. However, both TTA and pH were found to have insignificant associations with TSS and alcohol.

When presenting low alcoholic beverages, producers tend to mention their pH as an indication of character. The importance of acidity cannot be understated as it contributes to freshness and taste, acts as a preserving agent, and notably helps with microbial stability. When a pH measures above 3.8, many microorganisms can more easily proliferate, meaning there can be challenges with microbial stability in the final product during industrial processing; therefore, the lower the pH, the more desirable and safe the beverage is for consumption [36].

Co-culturing yeasts is ideal in the fermentation process of some low-alcoholic beverages. High alcohol levels can have several detrimental effects on some alcoholic beverages, including heightening the sensations of bitterness, astringency, and hotness, as well as obscuring some volatile aromatic constituents [37]. When the ethanol concentration is too high, fermentation may become halted or unresponsive [38]. High-alcohol beverages are well known for their harmful physiological as well as psychological impacts on the well-being of individuals [39].

To prevent a sugar increase in grapes and reduce ethanol in some alcoholic beverages, several viticultural and engineering solutions have been developed in this context. These solutions, however, can be costly and have a negative impact on the end product’s organoleptic quality. To minimise the alcohol concentration, inexpensive and straightforward methods are used such as early harvesting, which is the lowest impact approach to produce beverages with lower alcohol levels; the addition of water prior to fermentation in order to dilute high sugar levels, which is another method widely used; and lastly, the utilisation of appropriate wine yeasts that are capable of yielding lower amounts of alcohol during fermentation is of high interest, as it does not require additional labour, equipment, or handling [40].

*Non-Saccharomyces* yeasts have been found to have an impact on the composition, flavour, and sensory characteristics of beverages in several studies [41]. In addition, these yeasts have vital characteristics that could be utilised to potentially reduce ethanol content. In comparison to *Saccharomyces cerevisiae*, numerous *non-Saccharomyces* yeasts exhibit several respirofermentative regulatory mechanisms. Furthermore, several *non-Saccharomyces* yeasts produce less ethanol and have less fermentative efficiency than *S. cerevisiae* during the fermentation process [42]. When comparing the ethanol concentration produced with a single *S. cerevisiae* inoculum, recent investigations have revealed a decrease in ethanol when using yeast co-cultures [43]. As a result, *non-Saccharomyces* yeast fermentations with *S. cerevisiae* may be appealing for reducing ethanol while maintaining beverage quality.

## 5. Conclusions

The RSM models showed high accuracy in predicting optimum conditions for the selected parameters. The models for alcohol, pH, and TSS had R2 values of 0.79, 0.89, and 0.89, respectively, showing respective model accuracies of 79%, 89%, and 89%. RSM models were successfully validated by the constructed ANN with relatively high accuracy. The TSS, alcohol, and pH overall R^2^ values were 0.82, 0.98, and 0.88, respectively, signifying high validation accuracy and reliability in prediction.

For future research experiments, studies focusing on the beneficial effects of the consumption of bitter gourd, possible side effects that can occur with a diet high in bitter gourd, and controlled trials and assessments are needed for various products to be developed and introduced into the market.

## Figures and Tables

**Table 1:** RSM experimental design table and Factors selected for optimisation.

Run	X_1_ (Time)	X_2_ (Temperature)	X_3_ (Starter Culture Concentration)
1	120.00	25.00	1.00
2	72.00	45.11	3.00
3	24.00	40.00	1.00
4	120.00	40.00	1.00
5	72.00	32.50	3.00
6	24.00	40.00	5.00
7	24.00	25.00	5.00
8	120.00	25.00	5.00
9	72.00	32.50	3.00
10	152.73	32.50	3.00
11	72.00	32.50	3.00
12	72.00	19.89	3.00
13	72.00	32.50	0.36
14	72.00	32.50	6.36
15	0	32.50	3.00
16	72.00	32.50	3.00
17	120.00	40.00	5.00
18	72.00	32.50	3.00
19	24.00	25.00	1.00
20	72.00	32.50	3.00
Coded low (−1)	24.00	25.00	1.00
Coded mean (0)	72.00	32.5	3
Coded high (+1)	120.00	40.00	5.00

**Table 2:** Input factors and respective responses.

	Inputs			Responses			
Run	X1 (h)	X2 (°C)	X3 (*v*/*v*)	Y1 (°P)	Y2 (pH)	Y3 (% Lactic Acid)	Y4 (g/100 g)
1	120.00	25.00	1.00	4.50 d ± 0.25	3.80 cd ± 0.00	6.80 cd ± 0.20	3.30 c ± 0.06
2	72.00	45.11	3.00	11.70 i ± 0.00	4.00 h ± 0.06	7.10 ef ± 0.15	11.30 k ± 0.00
3	24.00	40.00	1.00	9.70 h ± 0.06	3.90 ef ± 0.00	6.70 bc ± 0.15	8.80 j ± 0.06
4	120.00	40.00	1.00	7.50 g ± 0.20	3.90 fg ± 0.06	7.30 fg ± 0.06	7.10 i ± 0.26
5	72.00	32.50	3.00	3.70 b ± 0.10	3.70 ab ± 0.00	6.30 a ± 0.26	3.90 e ± 0.06
6	24.00	40.00	5.00	9.60 h ± 0.17	3.90 ef ± 0.00	7.00 de ± 0.15	8.80 j ± 0.15
7	24.00	25.00	5.00	4.50 d ± 0.26	3.80 cd ± 0.10	6.70 bc ± 0.20	3.50 c ± 0.00
8	120.00	25.00	5.00	4.50 d ± 0.17	3.90 ef ± 0.00	7.60 gh ±0.17	3.50 c ± 0.17
9	72.00	32.50	3.00	4.80 e ± 0.06	3.70 ab ± 0.00	6.50 ab ± 0.00	3.90 e ± 0.06
10	152.73	32.50	3.00	4.90 e ± 0.10	4.00 gh ± 0.10	6.50 ab ± 0.00	5.20 g ± 0.10
11	72.00	32.50	3.00	3.90 bc ± 0.06	3.70 ab ± 0.00	7.00 de ± 0.06	3.90 de ± 0.00
12	72.00	19.89	3.00	4.70 de ± 0.12	3.90 ef ± 0.00	7.60 gh ± 0.10	3.70 d ± 0.17
13	72.00	32.50	0.00	0.90 a ± 0.10	3.60 a ± 0.06	6.70 bc ± 0.10	2.80 a ± 0.06
14	72.00	32.50	6.36	4.00 c ± 0.15	3.80 c ± 0.06	6.50 ab ± 0.00	3.70 de ± 0.12
15	0.00	32.50	3.00	1.10 a ± 0.17	3.80 c ± 0.06	7.60 gh ± 0.17	3.00 b ± 0.06
16	72.00	32.50	3.00	3.60 b ± 0.06	3.70 ab ± 0.00	7.30 fg ±0.29	3.80 de ± 0.06
17	120.00	40.00	5.00	6.00 f ± 0.35	3.90 ef ± 0.00	7.50 g ± 0.12	5.60 h ± 0.06
18	72.00	32.50	3.00	3.70 b ± 0.00	3.70 ab ± 0.06	7.50 g ± 0.17	3.80 de ± 0.10
19	24.00	25.00	1.00	4.90 e ± 0.20	3.80 cd ±0.00	7.80 i ± 0.15	4.80 f ± 0.12
20	72.00	32.50	3.00	3.70 b ± 0.20	3.80 c ± 0.06	6.60 bc ± 0.12	3.80 de ± 0.10

X_1_—fermentation time; X_2_—fermentation temperature; X_3_—starter culture concentration; Y_1_—alcohol; Y_2_—pH; Y_3_—TTA; Y_4_—TSS; measurements with the same letters (a–k) did not show significant differences.

**Table 3:** Fit statistics of the quadratic model for responses and analysis of variance (ANOVA) of alcohol (Y_1_), pH (Y_2_), TTA (Y_3_), and TSS (Y_4_) quadratic models.

Source	R^2^	Adjusted R^2^	F-Value	Lack of Fit	*p*-Value
Y_1_—Alcohol	0.79	0.60	4.20	F-value = 27.16, *p* = 0.00	0.02
Y_2_—pH	0.89	0.79	9.13	F-value = 1.94, *p* = 0.24	0.00
Y_3_—TTA	0.40	−0.14	0.75	F-value = 1.17, *p* = 0.43	0.67
Y_4_—TSS	0.89	0.79	8.96	F-value = 740.46, *p* = 0.00	0.00

**Table 4:** Training, validation, and test data.

	Alcohol		pH		TTA		TSS	
	MSE	R^2^	MSE	R^2^	MSE	R^2^	MSE	R^2^
Training (70%)	0.02	0.99	0.02	0.95	0.00	0.82	0.00	1.00
Validation (15%)	1.00	0.99	0.16	0.70	0.01	0.31	0.05	0.72
Testing (15%)	0.16	0.99	1.00	0.81	0.09	0.98	0.03	0.20
Overall	0.32	0.98	0.00	0.88	0.28	0.56	1.00	0.82

## Data Availability

The data that support the findings of this study are available from the corresponding author upon reasonable request. The authors confirmed that all the plant experiments were in compliance with relevant institutional, national, and international guidelines and legislation.
